# Exploring the association of physical activity on cognitive function in older adults from observational and genetic insights: a combined NHANES and Mendelian randomization study

**DOI:** 10.3389/fnagi.2024.1418455

**Published:** 2024-07-03

**Authors:** Hai-yan Liu, Yi-Jing Zhang, Wen-you Zhang

**Affiliations:** ^1^Department of Anesthesiology, The First Affiliated Hospital of Wenzhou Medical University, Wenzhou, Zhejiang, China; ^2^Department of Obstetrics, The First Affiliated Hospital of Wenzhou Medical University, Wenzhou, Zhejiang, China

**Keywords:** physical activity, cognitive function, older adults, NHANES, Mendelian randomization

## Abstract

**Background:**

Cognitive function (*CF*) deterioration is a pressing concern in geriatric research. This study aimed to explore the relationship between physical activity (PA) and *CF* in older adults.

**Methods:**

This study adopted a dual approach, employing both observational and genetic approaches through data from the National Health and Nutrition Examination Survey (NHANES) 2011–2014 and Mendelian Randomization (MR) analysis. For the NHANES component, PA levels were evaluated using the Global Physical Activity Questionnaire, and *CF* was assessed via standardized tests. Multivariate regression, threshold effect analysis, smoothing curve fitting, and subgroup analyses were conducted to examine the association between PA and *CF.* In parallel, MR methods, using genetic variants as instrumental variables, assessed the causal impact of PA on *CF* and related conditions such as Alzheimer’s disease and dementia.

**Results:**

Observational findings from NHANES demonstrated a positive correlation between PA and *CF*, notably among female participants. The detailed analysis identified specific thresholds of PA that correlate with cognitive enhancements. However, MR results did not support a significant causal relationship between PA and *CF* or dementia-related outcomes, indicating an absence of a direct genetic basis for the observational associations.

**Conclusion:**

Although observational data from NHANES suggest that PA is positively associated with *CF* in older adults, particularly among women, MR analysis did not confirm these findings as causally related. The discrepancy highlights the complexity of the PA-*CF* relationship and underscores the need for further research. These results emphasize the potential of PA as a modifiable risk factor for *CF*, though causal effects remain to be definitively established.

## 1 Introduction

The demographic trend toward a more aged global population has intensified the urgency to address cognitive decline and dementia among older adults (Petersen et al., 2014; Rabin et al., 2017; Chowdhary et al., 2021). As cognitive function (*CF*) is integral to maintaining autonomy, decision-making, and overall quality of life, the escalation in cognitive impairments presents a critical public health challenge (Rabin et al., 2017; Dolphin et al., 2024). This growing prevalence not only profoundly affects individuals but also places a considerable burden on families, healthcare systems, and societal resources (GBD 2015 Neurological Disorders Collaborator Group, 2017; Nandi et al., 2022). Therefore, effective interventions to combat the adverse effects of aging on *CF* and preserve mental acuity in the elderly are urgently needed.

Physical activity (PA) emerges as a promising non-pharmacological intervention with the potential to significantly enhance cognitive health (Raji et al., 2024). Beyond general health promotion, PA has been identified as a key factor in combating cognitive decline (Palta et al., 2019; Saint-Maurice et al., 2020; Raji et al., 2024). The extant research underscores that regular PA not only improves mental well-being but also acts as a preventive measure against the deterioration of *CF* (Li et al., 2023; Wang H, et al., 2024). However, comprehensive exploration of the relationship between PA and *CF* is limited, mostly due to the limited number of studies with large sample sizes and a relative dearth of research on causal correlations. Therefore, this necessitates further investigation to understand the underlying mechanisms and effects.

Although prior studies have recognized the positive impact of PA on cognitive well-being (Jehu et al., 2024; Lim et al., 2024; Mellow et al., 2024; Wang W, et al., 2024), the specific association by which PA influences *CF* and the causal relationships remain insufficiently understood. To address these limitations, our study adopts a pioneering approach by combining comprehensive cross-sectional data from the National Health and Nutrition Examination Survey (NHANES) 2011–2014 with Mendelian Randomization (MR) data from the UK Biobank. This innovative integration leverages NHANES’s extensive demographic, health, and nutritional data, and MR’s capacity to provide causality insights using genetic variants as instrumental variables (George and Shah, 2003; Md, 2009). Furthermore, Mendelian Randomization (MR) can elucidate causal relationships that are challenging to ascertain in traditional observational studies due to potential confounding factors and reverse causation (Md, 2009).

By amalgamating the detailed, population-wide insights from NHANES with the robust, causality-inferring framework of MR, our study aims to provide a more nuanced understanding of how PA influences *CF.* This combined approach allows us to transcend the limitations of traditional cross-sectional studies, potentially leading to more precise public health recommendations and the development of targeted, effective cognitive preservation strategies for the aging population. Our study hypothesizes that: Higher levels of PA are positively associated with better *CF* in older adults. MR analysis will provide evidence on the causality of this relationship.

## 2 Materials and methods

The NHANES dataset provides comprehensive cross-sectional data, including demographic, health, and nutritional information, which allows us to examine the association between PA and *CF* in a broad, real-world context. This dataset is used to assess the correlation between PA and *CF* among different demographic groups, providing valuable insights into the potential public health impact of PA. On the other hand, the MR analysis leverages genetic variants as instrumental variables to explore the causal relationship between PA and *CF.* By using data from the British Biobank, MR analysis helps to infer causality and reduce the bias inherent in observational studies. This approach allows us to address the limitations of NHANES, such as confounding factors and reverse causation, by providing a robust framework for causal inference. The combination of these two datasets enables a more comprehensive investigation into the relationship between PA and *CF.* While NHANES offers extensive observational data, MR provides the tools to establish causality. Together, they complement each other, enhancing our understanding of how PA impacts *CF* and potentially leading to more precise public health recommendations and effective cognitive preservation strategies for the aging population.

### 2.1 Methods of NHANES

#### 2.1.1 Inclusion and exclusion criteria of participants

**Figure 1:**
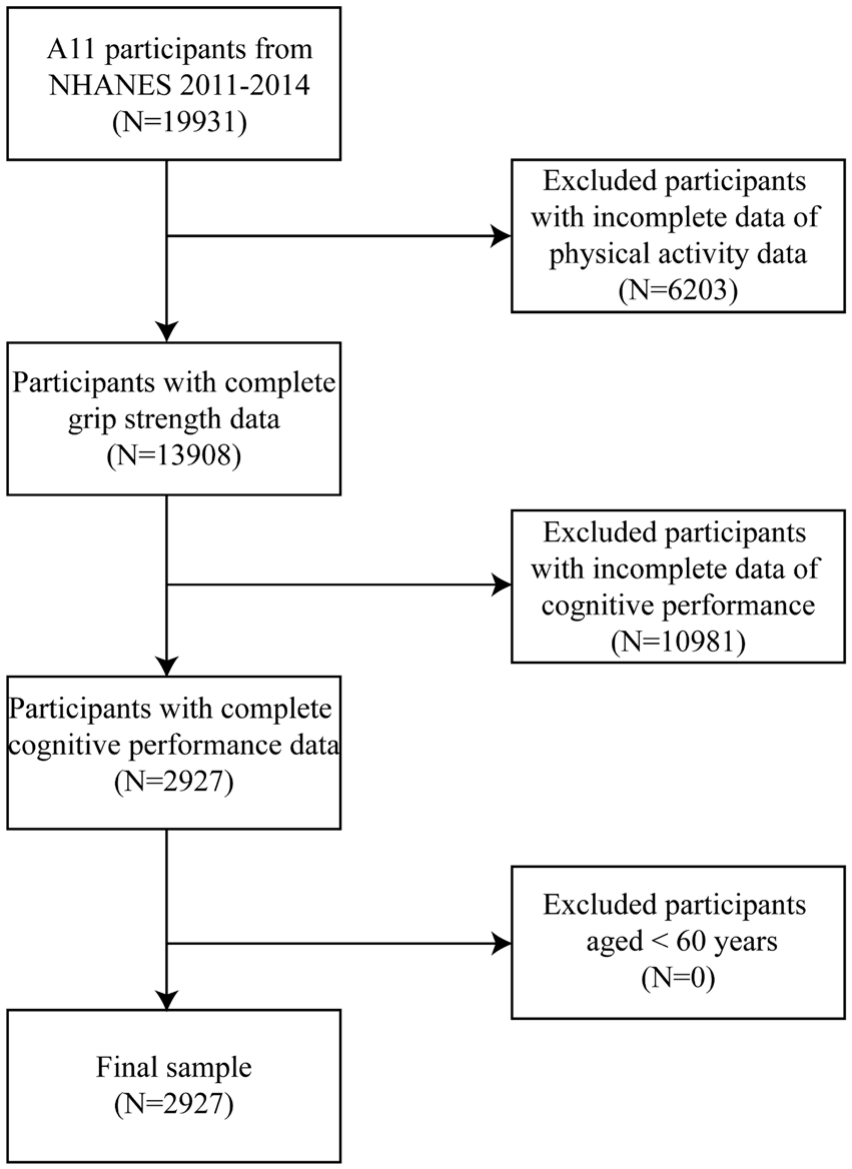
Inclusion and exclusion criteria of participants.

In the NHANES 2011–2014, the initial cohort comprised 19,931 participants. Exclusions were made for participants with incomplete PA records (*N* = 6,203) and those with incomplete *CF* data (*N* = 10,981), resulting in a study population of 2,927 individuals (Figure 1).

#### 2.1.2 Physical activity

The assessment of PA levels among participants was conducted utilizing data derived from the NHANES for the years 2011–2014. This period’s data collection employed the Global Physical Activity Questionnaire (GPAQ), encompassing a comprehensive range of questions designed to capture various dimensions of PA (Cleland et al., 2014). Specifically, the questionnaire solicited detailed information on daily activities, leisure time PA, and sedentary behaviors, facilitating a holistic evaluation of participants’ PA profiles (Cleland et al., 2014).

To quantify PA, we adopted methodologies consistent with those utilized in prior research, treating PA both as a continuous variable for analytical purposes and as a categorical variable for ease of interpretation and application to public health guidelines (You et al., 2023; Lei et al., 2024; Wang N, et al., 2024). The categorization of PA levels was grounded in the recommendations set forth by the United States Physical Activity Guidelines (Health USDo, 2008). Accordingly, a high level of PA was operationally defined as achieving 600 metabolic equivalent (MET)-minutes per week or more. Conversely, a low level of PA was defined as accumulating less than 600 MET-minutes per week (Health USDo, 2008). This dichotomous classification enables the examination of PA levels in relation to *CF* outcomes, aligning with established benchmarks for health-enhancing PA.

#### 2.1.3 Cognitive function

In this study, a series of standardized cognitive evaluations from the NHANES 2011–2014 data set were applied to gage *CF* in participants. These included the Consortium to Establish a Registry for Alzheimer’s Disease Word List utilized for evaluating both immediate recall over three trials and delayed recall after a set interval, aiming to measure short-term memory and learning retention (Morris et al., 1993). Additionally, verbal fluency and semantic memory were assessed through the Animal Fluency test, wherein participants were tasked with enumerating as many animal names as possible within a minute, reflecting on their capacity for rapid word generation within a specific category (Canning et al., 2004; Brody et al., 2019). The Digit Symbol Substitution Test was incorporated to examine executive function and processing speed, through a symbol-number matching task that tests attention, visual-motor coordination, and cognitive agility (Amaresha et al., 2014; Jaeger, 2018). To normalize the diverse cognitive domain scores for comparative analysis, Z-scores were calculated for each cognitive assessment. A composite score, representing the aggregate mean of these Z-scores, was subsequently computed to encapsulate an overarching measure of *CF* (Wilson, 2002).

#### 2.1.4 Covariate

In our study, we conscientiously incorporated a selection of covariates to control for potential confounding effects on the association between PA and *CF* (Jia et al., 2024; Wang X L, et al., 2024). These covariates encompassed key demographic factors (gender, race, age, education level), socio-economic status (Poverty Income Ratio, PIR), health and lifestyle indicators (body mass index, BMI; alcohol consumption frequency; waist circumference; sleep disorders), and health conditions (smoking status, diabetes, depressive symptoms). This careful selection was aimed at ensuring a comprehensive adjustment for variables known to influence cognitive outcomes, thereby enhancing the validity and reliability of our results.

#### 2.1.5 Statistical analysis

In our analysis, we focused on elucidating the relationship between PA and *CF* (including IR, DR, AF, and DSST), with a particular interest in gender-specific associations. Our investigation into the linear association between PA levels and *CF* was structured around three models of weighted multiple linear regression to accommodate varying degrees of adjustment. The initial model (Model 1) remained unadjusted. Model 2 introduced adjustments for a set of demographic variables, including race, age, Poverty Income Ratio (PIR), and education level. The most comprehensive model (Model 3) expanded upon this by adjusting for gender, race, age, education level, PIR, BMI, alcohol consumption frequency; waist circumference; sleep disorders, smoking status, diabetes, depressive symptoms. In addition, we employed smoothing curves and Generalized Additive Models (GAMs) to probe for potential nonlinear relationships between PA and *CF.* Lastly, threshold effect analysis was utilized in our study to enhance the analysis of the specific variations observed between PA and *CF.*

Statistical analyses were conducted using R software (version 4.2.3) and EmpowerStats (version 2.0), considering *p* < 0.05 as statistically significant. We represented categorical variables by percentages and continuous variables by their mean values ± standard deviations.

### 2.2 Mendelian randomization study

#### 2.2.1 Study design

In our investigation, we adopted a two-sample MR framework to elucidate the causal relationships between genetically inferred exposures (average physical activity (AVPA), moderate to vigorous physical activity (MVPA), and vigorous physical activity (VPA)) and health outcomes (*CF*, Alzheimer’s disease, and dementia). To uphold the integrity of our MR analysis, we strictly adhered to the established triangulation principles: (i) The instrumental genetic variants must exhibit robust associations with the exposures under consideration; (ii) These variants must remain unassociated with potential confounding variables to avoid spurious correlations; and (iii) The influence of these variants on the health outcomes should be mediated exclusively through the stipulated exposures (Emdin et al., 2017).

**Figure 2:**
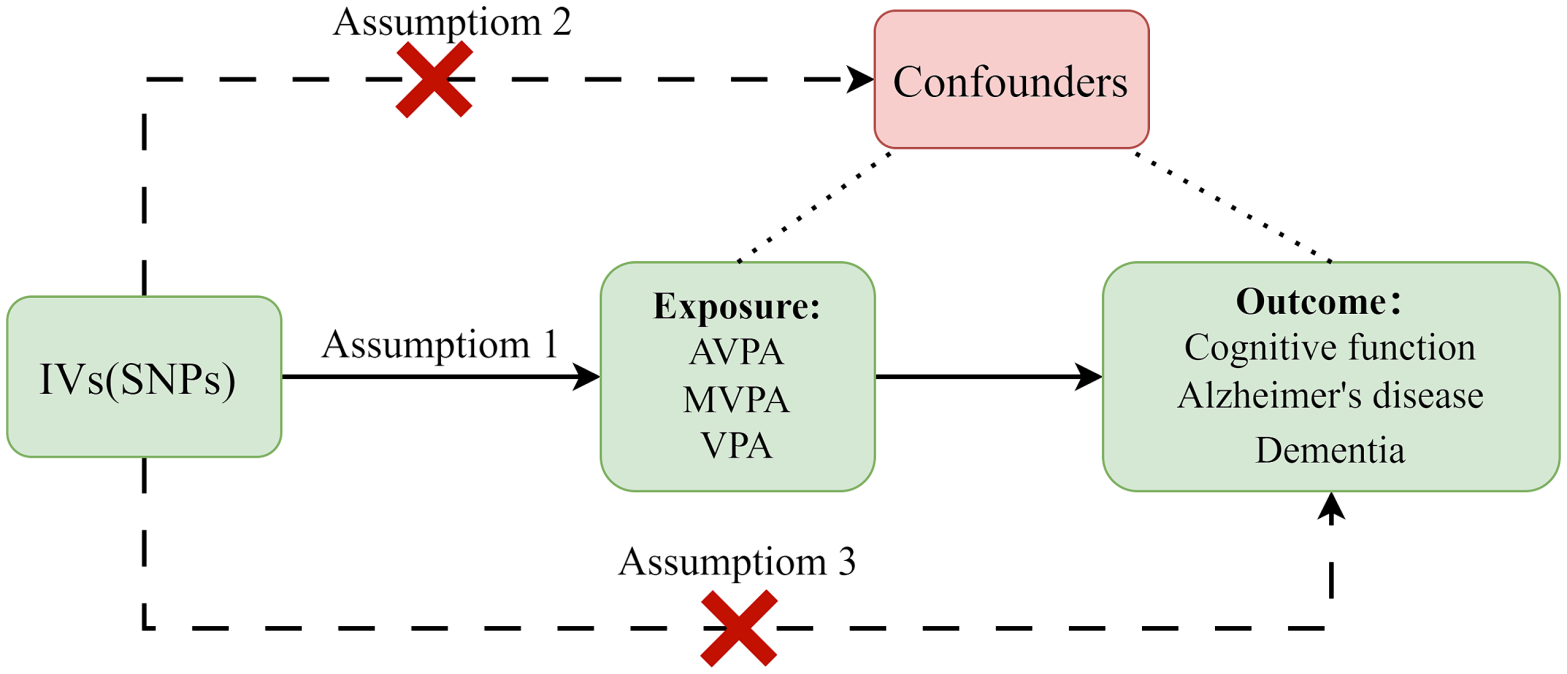
Principles of Mendelian randomization and assumptions. Assumption 1: The instrumental variables must be significantly associated with exposures. Assumption 2: They should not be correlated with any confounders. Assumption 3: Their impact on outcomes should be exclusively mediated through exposures. IVs, instrumental variables; SNPs, single nucleotide polymorphisms; AVPA, average physical activity; MVPA, moderate to vigorous physical activity; VPA, vigorous physical activity.

Our methodology, depicted in Figure 2, utilizes single nucleotide polymorphisms (SNPs) associated with PA levels as instrumental variables (IVs), thus leveraging comprehensive data from genome-wide association studies (GWAS). This strategy mitigates biases inherent in conventional observational studies, offering a more reliable inference of causal relationships.

#### 2.2.2 Genetic instruments selection

AVPA was evaluated using summary statistics from a GWAS analyzing accelerometer-derived PA metrics from approximately 100,000 UK Biobank participants (Klimentidis et al., 2018). Participants were equipped with the Axivity AX3, a wrist-worn triaxial accelerometer, programmed to collect data over a continuous seven-day period. Inclusion criteria mandated a minimum of 3 days (72 h) of data, with requisite activity recorded across all 24-h intervals. Exclusion criteria were applied to participants with insufficient data, uncalibrated devices, or those missing comprehensive daily activity cycles. Additionally, periods of non-wear, identified by stationary episodes exceeding 60 min with minimal deviation across all axes (<13 mg), were algorithmically imputed (Doherty et al., 2017). Unlike NHANES, which assesses PA based on self-reported questionnaires, MR analysis utilizes data from accelerometers (Chudyk et al., 2017).

The analytical model considered three primary metrics derived from the accelerometer data, encompassing a spectrum from 3 to 7 days of wear: Rabin et al. (2017). Average physical activity (AVPA): This metric accounts for all non-zero values of acceleration (Chowdhary et al., 2021). Moderate to vigorous physical Activity (MVPA): This is defined as the proportion of accelerations within the range of >100 mg to <425 mg (Petersen et al., 2014). Vigorous physical activity (VPA): This is defined as the proportion of accelerations exceeding 425 mg, up to 2000 mg (Klimentidis et al., 2018).

These metrics were refined through additional GWAS using the BGENIE software, focusing on decomposed acceleration signals from the UK Biobank (Bycroft et al., 2018). For the MR study, SNPs with strong associations with PA (genome-wide significance threshold *p* < 5×10^−8^) were utilized as IVs (Vaucher et al., 2018). We ensured IV independence by setting the linkage disequilibrium correlation coefficient to r^2^ < 0.001 and selecting a clumping window size of more than 10,000 kb.

To harmonize exposure and outcome data, SNPs that were missing, palindromic, incompatible, or directly related to outcomes were removed. To mitigate weak instrument bias, we calculated the F-statistic for each IV using the formula: $F_{\mathrm{exposure}}=\frac{{\mathrm{Beta}}_{\mathrm{exposure}}^{2}}{{SE}_{\mathrm{exposure}}^{2}}$ to evaluate IV strength (Lawlor et al., 2008). IVs with F-statistics under 10 were excluded to avoid biases from weak IVs, ensuring robustness in our MR methodology (Pierce et al., 2011).

#### 2.2.3 Summary dataset of outcome

Our study’s outcome variables were sourced from the comprehensive 2022 *CF* and Alzheimer’s disease datasets, under the GWAS identifiers ieu-b-4837 and ieu-b-5067, respectively, as provided by the IEU OpenGWAS project (2023a, 2023b). The dataset includes information from 9,997 and 488,285 European participants, respectively. Additionally, dementia-related outcomes were extracted from the 2021 FinnGen consortium dataset, which includes data from 7,284 cases (n case) and 209,487 controls (n control), offering extensive insights into the genetic underpinnings of dementia and related pathologies (FinnGen consortium dataset, 2023).

#### 2.2.4 Statistical analysis

In our MR analysis, we used the Inverse Variance Weighted (IVW) method as the primary analytical tool (Burgess et al., 2013). The selection between the fixed effect IVW (IVW-FE) and random effect IVW (IVW-RE) models was determined based on the Cochrane Q heterogeneity test outcomes. The IVW-RE model, known for its more conservative estimations, was utilized in scenarios where significant heterogeneity was present (*p* < 0.05). In contrast, the IVW-FE model was adopted in cases lacking such heterogeneity (Greco et al., 2015).

To bolster the credibility of our findings, three additional analytical methods were used: weighted median, simple mode, and weighted mode. The weighted median approach assumes that fewer than 50% of the IVs are affected by horizontal pleiotropy (Bowden et al., 2016). Crucial sensitivity analyses included MR-Egger regression intercept tests to evaluate pleiotropy (Burgess et al., 2017). Additionally, a leave-one-out analysis was implemented to ensure the stability of our MR findings. This involved sequentially removing each SNP from the instrumental variables and recalculating the causal effect without that specific SNP, aiming to identify any single SNP’s disproportionate influence on the MR estimate (Burgess et al., 2017). Only exposure-outcome pairs that showed a consistent direction across all MR methods and a significant result in all analysis were confirmed to be causal.

Statistical significance was set at *p* < 0.05, with odds ratio (OR), standard error (SE), and 95% confidence intervals (95% CI) presenting the results of causal associations. These analyses were conducted using the “TwoSampleMR” package (version 0.5.6; Liang et al., 2018) in R software (version 4.2.3).

## 3 Results

### 3.1 Results of NHANES

#### 3.1.1 Basic information on the study participants

Table 1 illustrates the demographic characteristics of the study participants. Among the 2,927 participants, 1,330 were males and 1,597 were females, with an average age of 69.20 ± 6.65 years. Significant demographic disparities were observed between genders in terms of age, education level, and the ratio of family income to poverty. Additionally, there were notable differences between males and females regarding the prevalence of diabetes, symptoms of depression, waist circumference, frequency of alcohol consumption, smoking status, and levels of PA. Cognitive performance also significantly differed between participants with high and low levels of PA.

**Table 1:** Basic information on the study population.

Categorical scalar (%)	All (*N* = 2,927)	Male (*N* = 1,330)	Female (*N* = 1,597)	*p-*value
PA level (MET-min/week)				<0.0001
High level	51.20	42.50	54.05	
Low level	48.80	57.50	45.95	
Race				0.5034
Mexican American	3.39	3.63	3.18	
Other Hispanic	3.65	3.40	3.87	
Non-Hispanic White	79.45	80.20	78.83	
Non-Hispanic Black	8.47	7.60	9.20	
Other	5.03	5.17	4.92	
Education level				<0.0001
< 9th grade	5.70	6.17	5.31	
9-11th grade	10.27	9.43	10.96	
High school graduate/GED	22.17	19.79	24.16	
College or AA degree	31.30	27.96	34.08	
College graduate or above	30.56	36.65	25.49	
Smoking status				<0.0001
Yes	50.27	60.83	41.47	
No	49.73	39.17	58.53	
Diabetes				0.0121
Yes	19.42	21.69	17.53	
No	76.50	73.99	78.60	
Boundary	4.08	4.32	3.87	
Depressive symptoms				0.0001
Yes	8.50	6.30	10.32	
No	91.50	93.70	89.68	
Sleep disorders				<0.0001
Yes	31.53	24.68	37.23	
No	68.47	75.32	62.77	
Continuous variables (M ± SD)				
Age(year)	69.20 ± 6.65	68.84 ± 6.54	69.50 ± 6.73	0.0072
PIR	3.08 ± 1.53	3.29 ± 1.51	2.91 ± 1.54	<0.0001
Alcohol frequency (times/year)	4.88 ± 22.20	7.25 ± 32.60	2.91 ± 3.14	<0.0001
BMI (kg/m^2^)	29.06 ± 6.24	28.92 ± 5.63	29.18 ± 6.70	0.2727
Waist circumference(cm)	102.39 ± 14.30	106.12 ± 13.94	99.28 ± 13.86	<0.0001
IR	0.18 ± 0.97	0.03 ± 0.92	0.30 ± 0.99	<0.0001
DR	0.14 ± 0.99	−0.00 ± 0.96	0.25 ± 1.00	<0.0001
AF	0.28 ± 1.04	0.33 ± 1.06	0.23 ± 1.02	0.0057
DSST	0.36 ± 0.97	0.26 ± 0.90	0.45 ± 1.02	<0.0001
Cognitive function(score)	0.24 ± 0.79	0.15 ± 0.75	0.31 ± 0.82	<0.0001

PA, physical activity; BMI, body mass index; PIR, Ratio of family income to poverty; IR, Word list learning trials (immediate recall); DR, Word list learning trials (delayed recall); AF, Animal fluency; DSST, Digit symbol substitution test. Continuous variables are described using mean ± standard deviation (M ± SD), while categorical variables are represented by percentages (%). Individuals with a score > 9 on the Patient Health Questionnaire [PHQ-9] were considered to have depressive symptoms. High level of physical activity was defined as ≥ 600 MET-min/week and low level of physical activity was defined as < 600 MET-min/week.

#### 3.1.2 Association between PA and *CF* stratified by gender

As illustrated in Table 2, PA was analyzed as both a continuous and categorical variable (Low level and High level) in relation to *CF*, with stratification by gender through multivariate regression analysis. Overall, a positive correlation between PA and *CF* was identified (*p* = 0.0248), with the High-level PA group exhibiting significantly superior *CF* compared to the Low-level PA group (*p* < 0.001). Upon gender stratification, a positive correlation between PA and *CF* was observed exclusively in females (*p* < 0.0142).

**Table 2:** Association between PA and *CF* stratified by gender.

*β* (95% CI)			
	Male	Female	Total
Model 1			
PA(MET-min/week)	0.00 (0.00, 0.00) <0.0001	0.00 (0.00, 0.00) <0.0001	0.00 (0.00, 0.00) <0.0001
Low level	0 (reference)	0 (reference)	0 (reference)
High level	0.30 (0.23, 0.38) <0.0001	0.36 (0.28, 0.44) <0.0001	0.34 (0.28, 0.39) <0.0001
P for trend	<0.001	<0.001	<0.001
Model 2			
PA(MET-min/week)	0.00 (−0.00, 0.00) 0.1791	0.00 (0.00, 0.00) 0.0099	0.00 (0.00, 0.00) 0.0090
Low level	0 (reference)	0 (reference)	0 (reference)
High level	0.11 (0.05, 0.17) 0.0005	0.18 (0.12, 0.25) <0.0001	0.15 (0.11, 0.20) <0.0001
P for trend	<0.001	<0.001	<0.001
Model 3			
PA(MET-min/week)	0.00 (−0.00, 0.00) 0.2718	0.00 (0.00, 0.00) 0.0142	0.00 (0.00, 0.00) 0.0248
Low level	0 (reference)	0 (reference)	0 (reference)
High level	0.11 (0.05, 0.17) 0.0009	0.18 (0.11, 0.24) <0.0001	0.15 (0.10, 0.19) <0.0001
P for trend	<0.001	<0.001	<0.001

PA, physical activity; CF, cognitive function; β, beta; CI, confidence intervals. Model 1: Unadjusted Variables. Model 2: Race, age, education level, ratio of family income to poverty were adjusted. Model 3: Race, age, education level, ratio of family income to poverty, alcohol frequency, waist circumference, BMI, smoking status, diabetes, depressive symptom, were adjusted.

Additionally, when analyzing PA against individual cognitive assessments (IR, DR, AF, DSST) stratified by gender (see Supplementary Tables S1–S4 for detailed results), we found varied correlations. Generally, PA showed no significant correlation with IR, DR, and DSST, except for AF. However, participants in the High PA group scored significantly higher on all cognitive tests compared to those in the Low PA group.

Upon gender stratification, both male and female PA levels were positively correlated with AF. Additionally, female PA levels were positively correlated with DSST. Specifically, females with high PA levels significantly outperformed those with low PA levels in all four cognitive tests. In contrast, males with high PA levels only showed significant improvement in AF and DSST.

#### 3.1.3 Smoothing curves and threshold effect analysis

**Figure 3:**
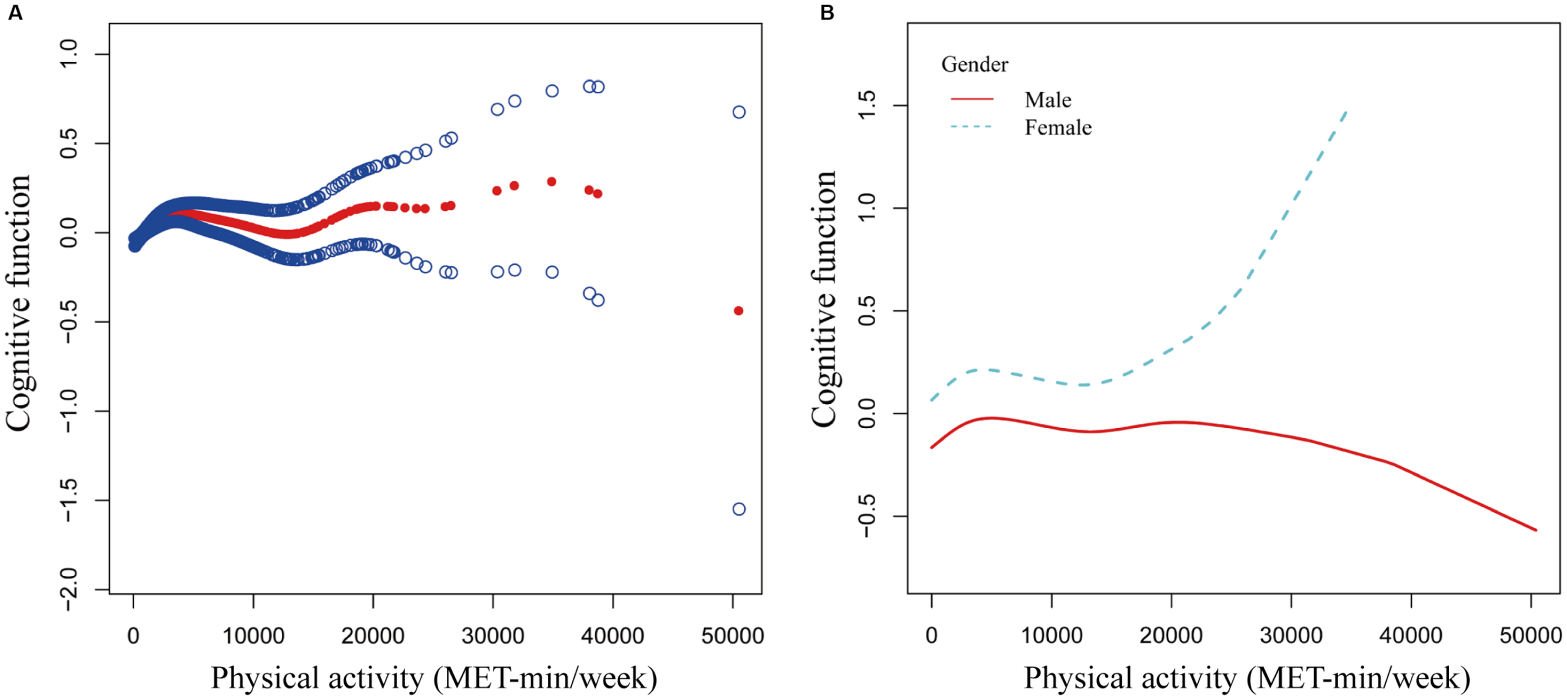
Smoothing curves. **(A)** PA and overall *CF.* **(B)** PA and overall *CF* stratified by gender.

As demonstrated in Table 3 and Figure 3, threshold effect analysis and smoothing curve fitting were applied to explore the relationship between PA and *CF.* The analysis identified inflection points at 2100 for male PA, and 120 for both female PA and overall PA. For males, PA exhibited a positive correlation with *CF* at levels below 2,100, with no significant correlation observed above this threshold. In females, PA positively correlated with *CF* when below 120, but not at higher levels. Similarly, for the general analysis, PA showed a positive correlation with *CF* when under 120, with no significant relationship beyond this point. The detailed smoothing curve fittings for PA and *CF* interactions are provided in Figure 3.

**Table 3:** Threshold effect analysis of PA on overall *CF.*

	*β* (95% CI)		
	Male	Female	Total
One-line linear regression model	0.00 (−0.00, 0.00) 0.2718	0.00 (0.00, 0.00) 0.0142	0.00 (0.00, 0.00) 0.0248
Two-piecewise linear regression model			
Inflection point (K)	2,100	120	120
PA < K(MET-min/week)	0.00 (0.00, 0.00) <0.0001	0.00 (0.00, 0.00) <0.0001	0.00 (0.00, 0.00) <0.0001
PA ≥ K(MET-min/week)	−0.00 (−0.00, 0.00) 0.0685	0.00 (−0.00, 0.00) 0.7229	−0.00 (−0.00, 0.00) 0.6768
Log-likelihood ratio	<0.001	<0.001	<0.001

PA, physical activity; CF, cognitive function; β, beta; CI, confidence intervals.

Additionally, threshold effect analyses and smoothing curve fittings were conducted for PA against the cognitive assessments of Immediate Recall, Delayed Recall, Animal Fluency, and the Digit Symbol Substitution Test. The results indicated significant nonlinear relationships between PA and all cognitive tests across participants, except for a non-significant relationship between PA and Immediate Recall in male participants. Detailed findings are provided in Supplementary Tables S5–S8 and Supplementary Figures S1, S2.

### 3.2 Results of MR analysis

In our research, 5 to 18 SNPs were chosen as IVs for exposures. For an in-depth overview of these IVs utilized in MR analysis, please refer to Supplementary Table S9. The F-statistics of each IV varied, ranging from 29.972 to 55.256. Given the heterogeneity observed in MVPA and dementia as determined by Cochrane’s Q test (*p* < 0.05; detailed in Table 4), the IVW-RE model was chosen as the primary method for MR analysis. The MR results indicated no causal link of PA (AVPA, MVPA, and VPA) on *CF*, Alzheimer’s disease, and dementia. Specifically, for AVPA, the odds ratio varied from 0.973 (95%CI: 0.909,1.042, *p* = 0.436) for dementia to 1.014 (95%CI: 0.961, 1.070, *p* = 0.618) for *CF*; for MVPA, the odds ratio varied from 0.882 (95%CI: 0.561, 1.385, *p* = 0.585) for *CF* to 1.002 (95%CI: 0.998, 1.005, *p* = 0.388) for Alzheimer’s disease; for VPA, the odds ratio varied from 0.420 (95%CI: 0.133, 1.328, *p* = 0.140) for *CF* to 1.801 (95%CI: 0.344, 9.433, *p* = 0.486) for dementia (Table 4). Other MR methods were finding no causal link either.

**Table 4:** Mendelian randomization analysis results.

Exposure	Outcome	Method	SNPs	MR		Heterogeneity		Pleiotropy		
				OR (95%CI)	*p*	Q	*p*	Egger_intercept	SE	*p*
AVPA	Cognitive function	IVW-FE	5	1.01 (0.96, 1.07)	0.62	1.56	0.82	0.02	0.03	0.64
		Weighted median		1.01 (0.95, 1.09)	0.71					
		Simple mode		1.01 (0.92, 1.10)	0.92					
		Weighted mode		1.01 (0.93, 1.09)	0.90					
MVPA		IVW-FE	18	0.88 (0.56, 1.39)	0.59	11.47	0.83	−0.01	0.02	0.83
		Weighted median		0.73 (0.39, 1.35)	0.31					
		Simple mode		1.28 (0.42, 3.96)	0.67					
		Weighted mode		0.62 (0.22, 1.77)	0.39					
VPA		IVW-FE	7	0.42 (0.13, 1.33)	0.14	3.43	0.75	−0.02	0.05	0.64
		Weighted median		0.56 (0.13, 2.34)	0.42					
		Simple mode		0.83 (0.09, 8.03)	0.88					
		Weighted mode		0.77 (0.09, 6.61)	0.82					
AVPA	Alzheimer’s disease	IVW-FE	7	1.00 (1.00, 1.00)	0.33	10.23	0.12	0.01	0.01	0.43
		Weighted median		1.00 (1.00, 1.00)	0.80					
		Simple mode		1.00 (1.00, 1.00)	0.23					
		Weighted mode		1.00 (0.99, 1.00)	0.90					
MVPA		IVW-FE	17	1.00 (0.99, 1.01)	0.39	7.36	0.97	−0.01	0.01	0.79
		Weighted median		1.00 (0.99, 1.01)	0.60					
		Simple mode		1.00 (0.99, 1.01)	0.75					
		Weighted mode		1.00 (0.99, 1.01)	0.85					
VPA		IVW-FE	7	0.99 (0.99, 1.00)	0.12	2.05	0.92	0.01	0.01	0.87
		Weighted median		0.99 (0.98, 1.00)	0.13					
		Simple mode		0.99 (0.98, 1.01)	0.32					
		Weighted mode		0.99 (0.98, 1.01)	0.26					
AVPA	Dementia	IVW-FE	8	0.97 (0.91, 1.04)	0.44	10.32	0.17	−0.02	0.05	0.65
		Weighted median		1.00 (0.92, 1.10)	0.98					
		Simple mode		1.04 (0.90, 1.19)	0.64					
		Weighted mode		1.03 (0.90, 1.17)	0.72					
MVPA		IVW-RE	17	0.99 (0.38, 2.47)	0.98	26.99	0.04	0.01	0.05	0.87
		Weighted median		0.74 (0.28, 1.97)	0.55					
		Simple mode		0.67 (0.13, 3.49)	0.65					
		Weighted mode		0.80 (0.17, 3.85)	0.79					
VPA		IVW-FE	7	1.80 (0.34, 9.43)	0.49	2.80	0.83	−0.07	0.06	0.29
		Weighted median		0.85 (0.11, 6.59)	0.88					
		Simple mode		0.82 (0.04, 17.95)	0.90					
		Weighted mode		0.79 (0.04, 16.04)	0.89					

AVPA, average physical activity; MVPA, moderate to vigorous physical activity; VPA, vigorous physical activity; IVW-FE, inverse variance weighted (fixed effect); IVW-RE, inverse variance weighted (multiplicative random effect); OR, odds ratio; SE, standard error; MR, Mendelian randomization; SNPs; single nucleotide polymorphisms.

Sensitivity analyses, including MR-Egger intercept tests, indicated that horizontal pleiotropy did not significantly impact the MR analysis (all *p* > 0.05; Table 4). Leave-one-out sensitivity analysis confirmed the robustness of the MR results, showing no significant distortion of outcomes after the removal of any single SNP (Supplementary Figures S3–S11).

## 4 Discussion

Our study aimed to investigate the relationship between PA and *CF* using both cross-sectional and MR analysis. The findings from our cross-sectional study indicate that, as a continuous variable, PA is positively associated with *CF* across the general cohort and specifically within the female subgroup, but not in males. Conversely, when analyzed as a categorical variable, higher levels of PA were consistently associated with superior *CF* compared to lower levels, across both genders. However, the absence of a causal relationship from our MR analysis between PA levels and *CF* challenges straightforward interpretations and signals that additional variables may be at play in cognitive health. This divergence emphasizes the intricacies of PA’s effects on cognition and underlines the necessity for broader exploratory efforts in this field.

Our NHANES cross-sectional analysis echoes existing studies in highlighting the link between PA and improved *CF* in older adults, reinforcing the protective role of PA against cognitive deterioration (Jung et al., 2023; Werneck et al., 2023; You et al., 2023; Zheng et al., 2023). Our study extends these findings by illustrating a gender-specific effect, particularly noting enhanced benefits in older women. Although estrogen, known for its neuroprotective properties, is considered an important hormone for cognitive health (Bagit et al., 2021; Raciti et al., 2023), postmenopausal women experience a natural decline in estrogen levels. Therefore, the improvement in cognitive health in older women through PA may be achieved via other pathways. To delve deeper into the potential mechanisms, some study consider the role of neurotrophic factors, particularly brain-derived neurotrophic factor (BDNF; Peng et al., 2010; Kim et al., 2011; Aaron and Piepmeier Jennifer, 2015). BDNF is a key player in neuroplasticity, neuroprotection, and neurogenesis, all of which are crucial for maintaining cognitive function (Peng et al., 2010; Aaron and Piepmeier Jennifer, 2015). PA has been shown to significantly increase BDNF levels, thereby enhancing cognitive health (Peng et al., 2010; Kim et al., 2011; Aaron and Piepmeier Jennifer, 2015). This effect is especially important for postmenopausal women who experience a decline in estrogen levels (Farrukh et al., 2023). Moreover, the intensity and type of PA can differentially impact BDNF levels. High-intensity interval training (HIIT) has been found to induce more significant and immediate increases in BDNF compared to aerobic exercise, likely due to the intense, intermittent nature of HIIT, which stimulates greater neurotrophic responses (Boyne et al., 2019). However, while single sessions of aerobic exercise may not elevate BDNF levels as dramatically as HIIT, regular aerobic exercise is believed to be more beneficial for BDNF over time, especially in older adults (Boyne et al., 2019; Farrukh et al., 2023). Given that older women tend to engage more in moderate-intensity physical activities (Li et al., 2017; Liao et al., 2021), they seem to experience more sustained BDNF improvement benefits. Additionally, the metabolic and inflammatory responses to PA might differ between genders (Ansdell et al., 2019; Gado et al., 2024). As inflammation and metabolism are closely linked to cognitive health, the more substantial reductions in inflammatory markers and improvements in metabolic profiles resulting from PA could translate into more pronounced cognitive benefits for women (Montero-Odasso et al., 2018; Tan et al., 2023). Furthermore, considering that women generally have higher body fat percentages than men, the metabolic benefits of PA, such as improved insulin sensitivity and lipid profiles, may play a more critical role in maintaining cognitive health in older women (Kullmann et al., 2022; Heni, 2024; Vicente-Gabriel et al., 2024). Further research is needed to explore and validate the underlying effect mechanisms in more detail.

On the other hand, our MR analysis did not demonstrate a causal relationship between PA and *CF*, diverging from some previous causal findings (Zhang et al., 2022; Cheval et al., 2023). Previous studies, such as those by Zhang et al. (2022) and Cheval et al. (2023), found significant causal links between PA and *CF* or Alzheimer’s disease using different methodologies and populations. Zhang et al. (2022) found that genetically predicted walking was associated with a reduced risk of Alzheimer’s disease, while no significant association was observed for overall activity, sedentary behavior, or moderate-intensity activity. The protective effect of walking on Alzheimer’s disease risk highlights the potential benefits of specific types of physical activity (Zhang et al., 2022). Cheval et al. (2023) reported significant associations suggesting that MVPA positively influenced cognitive function. However, no causal effect was found for average physical activity (Cheval et al., 2023).

This variance could be attributed to differences in data sources among MR studies. MR analysis rely heavily on the genetic variants chosen as instrumental variables for PA. For instance, genetic instruments derived from one population may not be entirely applicable to another due to differences in lifestyle, environmental exposures, or gene–environment interactions, leading to disparate outcomes (Wang N, et al., 2024). Furthermore, our study’s MR analysis relied on specific sets of SNPs as instrumental variables, which may differ from those used in previous studies. The selection of genetic instruments and the statistical power of the analysis can significantly impact the results. Zhang et al. (2022) and Cheval et al. (2023) employed different MR methodologies and sensitivity analyses, which could account for the discrepancies (Zhang et al., 2022; Cheval et al., 2023). Additionally, time and geographical differences within the datasets should be considered, especially in studies of cognition. The NHANES dataset provides cross-sectional data from the United States, while the MR analysis utilizes data from the UK Biobank. These datasets represent different populations with distinct lifestyle factors, environmental exposures, and healthcare systems, which could influence cognitive outcomes. Furthermore, advancements in technology and data collection methods over the years may also contribute to differences in findings. Ancestry and population mismatch are critical factors that cannot be dismissed, as genetic instruments may not have the same relevance or effect across diverse populations.

The principal strength of our research lies in the innovative use of NHANES 2011–2014 cross-sectional methods combined with MR methods, offering a comprehensive insight into the complex relationship between PA and *CF.* By leveraging the strengths of both methods, we can explore the association from different analytical perspectives. This innovative approach allows us to explore the PA-*CF* relationship at a lower cost, significantly reducing the time and financial resources typically required for large-scale randomized controlled trials or longitudinal studies. However, our study is not without limitations. First, the generalizability of our findings is limited. While NHANES is representative of the population in the US, MR study individuals are European. Thus, our results may not be applicable to populations in other countries or regions with different lifestyles, healthcare systems, or genetic backgrounds. Additionally, the multiracial nature of the NHANES database could contribute to the lack of causal associations observed. Differences in genetic backgrounds and environmental exposures across diverse populations may introduce variability that impacts the results. Moreover, one limitation of our study is the variation in PA measurement methods between the datasets used. NHANES assesses PA based on self-reported questionnaires, which may introduce recall bias and subjective interpretation, potentially making PA assessment less accurate compared to accelerometer measurements. Furthermore, the possibility of unidentified SNPs relevant to the study may affect the strength and clarity of the causal relationships we aimed to explore. Lastly, while we have attempted to control for various confounding factors, there may be unmeasured confounders that could affect the observed relationship between PA and *CF.* Factors such as diet, socioeconomic status, or other lifestyle habits could play a significant role in cognitive health and may not be fully accounted for in our analysis.

## 5 Conclusion

Our study reveals a positive association between PA and *CF* in older adults, particularly among women, based on data from NHANES 2011–2014. Despite these associations, the lack of a causal link established through MR suggests the complexity of this relationship and the need for further investigation. These findings underscore the potential of PA as a modifiable factor for enhancing cognitive health in the aging population, highlighting the importance of continued research in this area.

## Data availability statement

The original contributions presented in the study are included in the article/Supplementary material, further inquiries can be directed to the corresponding author.

## Ethics statement

All data included in this study obtained ethical approval from the respective institutions. Written informed consent was obtained in accordance with the national legislation and the institutional requirements.

## Author contributions

H-yL: Data curation, Formal analysis, Writing – original draft, Writing – review & editing. Y-JZ: Formal analysis, Investigation, Methodology, Writing – original draft, Writing – review & editing. W-yZ: Conceptualization, Supervision, Writing – original draft, Writing – review & editing.
