# Supplementary material for: Exploring the association of physical activity on cognitive function in older adults from observational and genetic insights: a combined NHANES and Mendelian randomization study
Source: Front Aging Neurosci. 2024 Jul 3;16:1418455. doi: 10.3389/fnagi.2024.1418455 (PMC11252077; doi:10.3389/fnagi.2024.1418455)
Supplement: Supplementary file 2 [file Data_Sheet_1.docx]

Supplementary Material

## Supplementary Figures


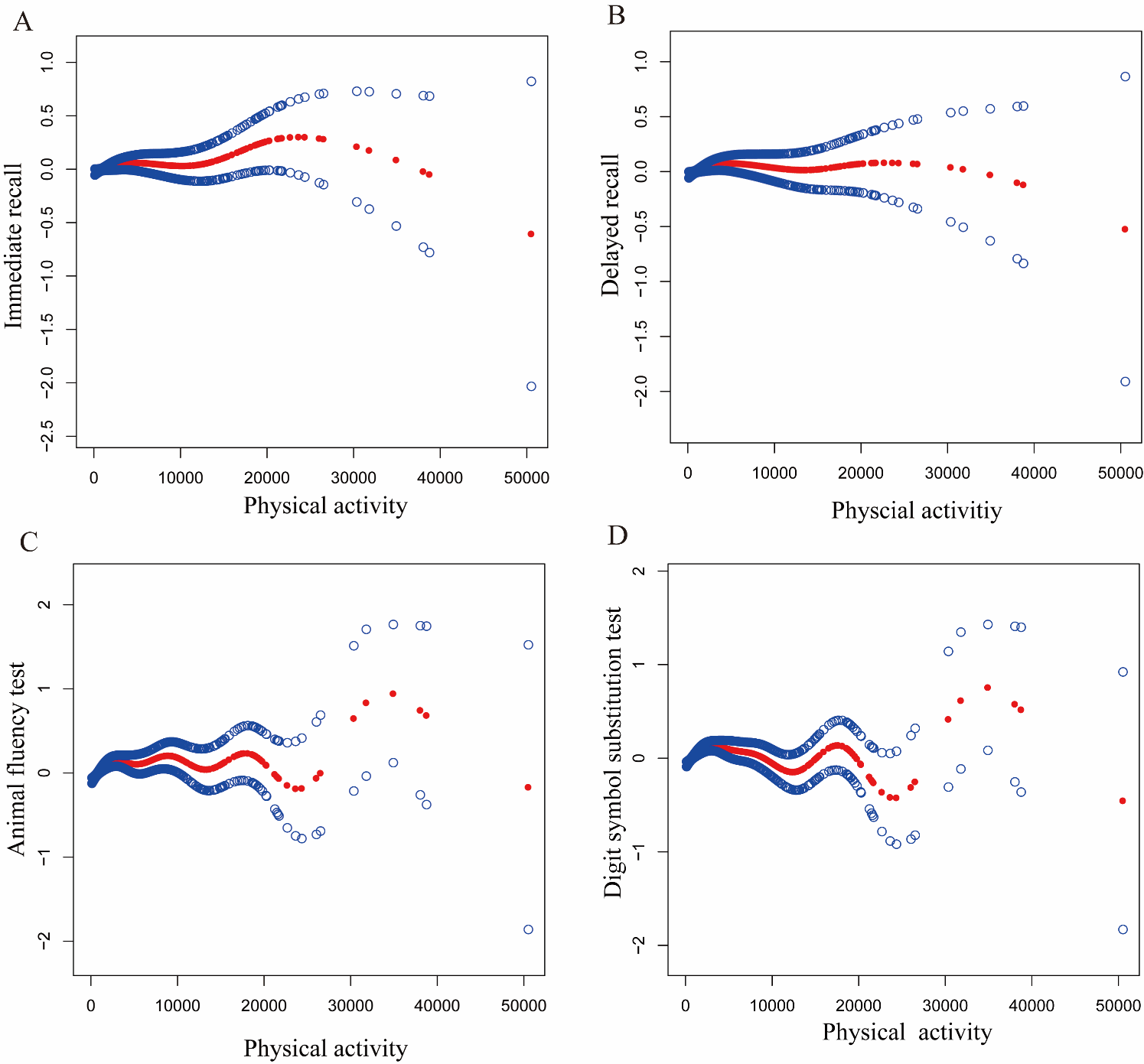


**Supplementary Figure S1.** Smoothed curves of PA and each cognitive test (A) Smoothed curve of PA and IR; (B) Smoothed curve of PA and DR; (C) Smoothed curve of PA and AF; (D) Smoothed curve of PA and DSST. PA: physical activity; IR: Word list learning trials (immediate recall); DR: Word list learning trials (delayed recall); AF: Animal fluency; DSST: Digit symbol substitution test.


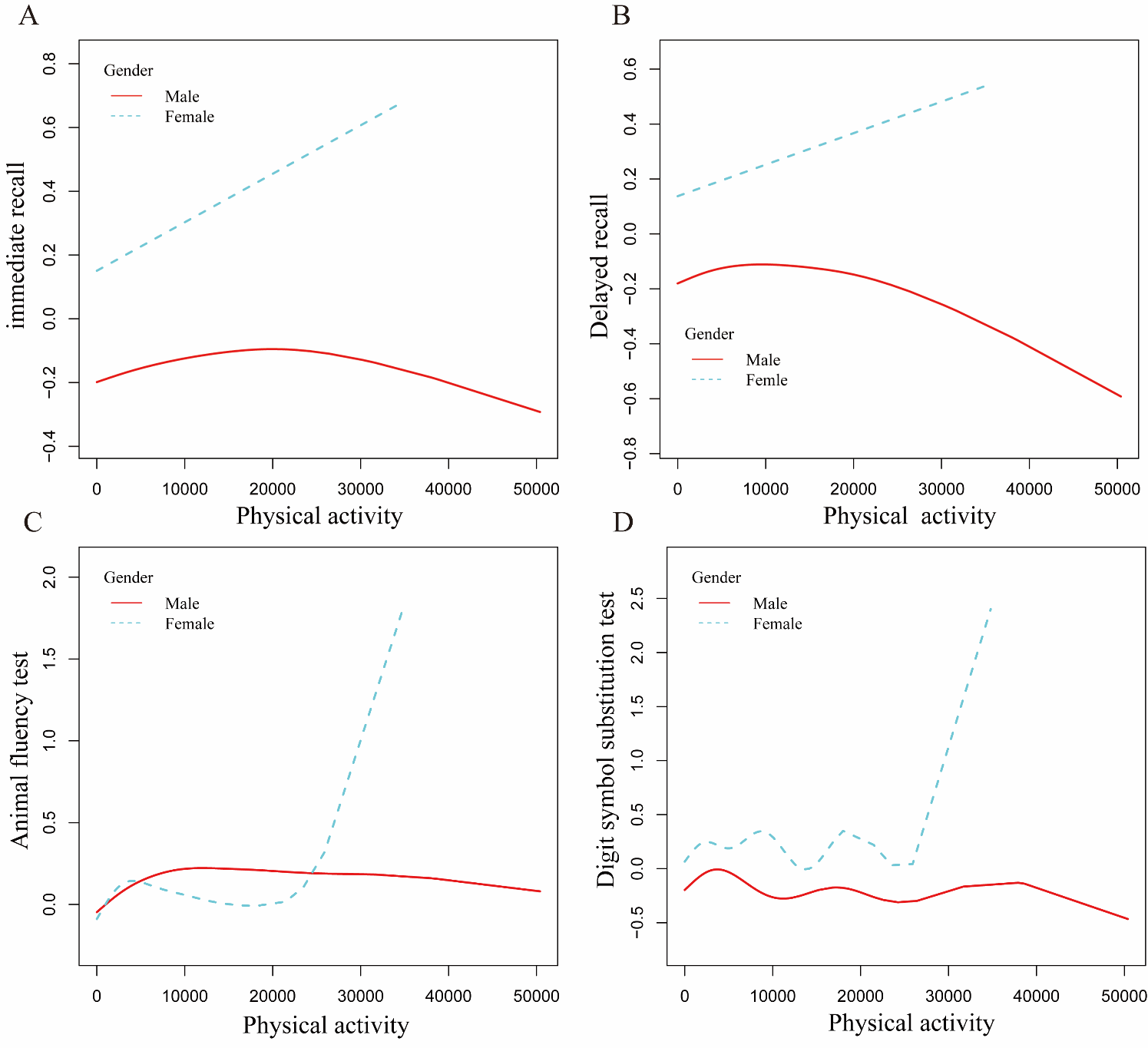


**Supplementary Figure S2.** Smoothed curves of PA and each cognitive test stratified by gender (A) Smoothed curve of PA and IR stratified by gender; (B) Smoothed curve of PA and DR stratified by gender; (C) Smoothed curve of PA and AF stratified by gender; (D) Smoothed curve of PA and DSST stratified by gender. PA: physical activity; IR: Word list learning trials (immediate recall); DR: Word list learning trials (delayed recall); AF: Animal fluency; DSST: Digit symbol substitution test.


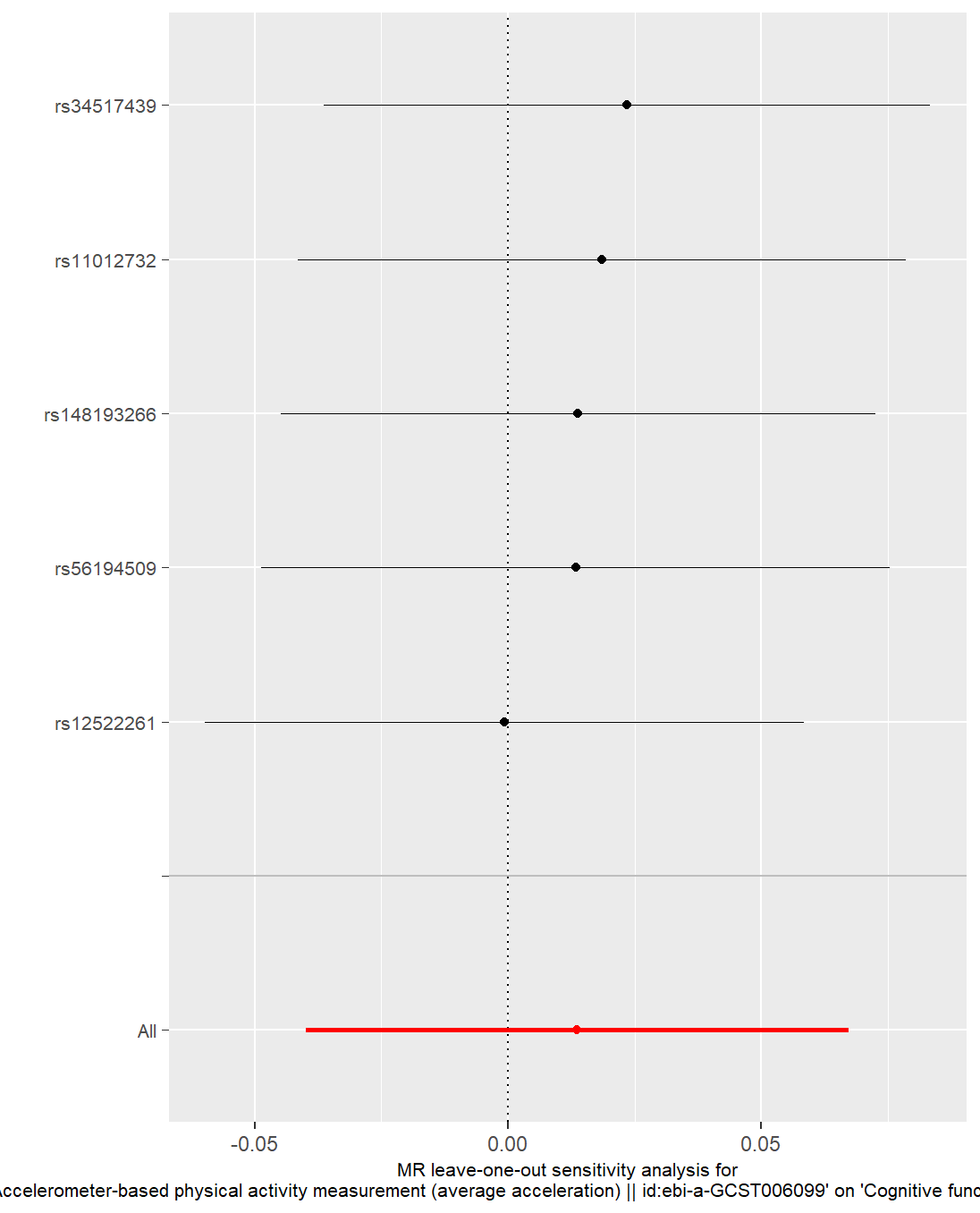


**Supplementary Figure S3.** Leave-one-out sensitivity analysis of average physical activity on cognitive function under genome-wide significance threshold instrumental variables.


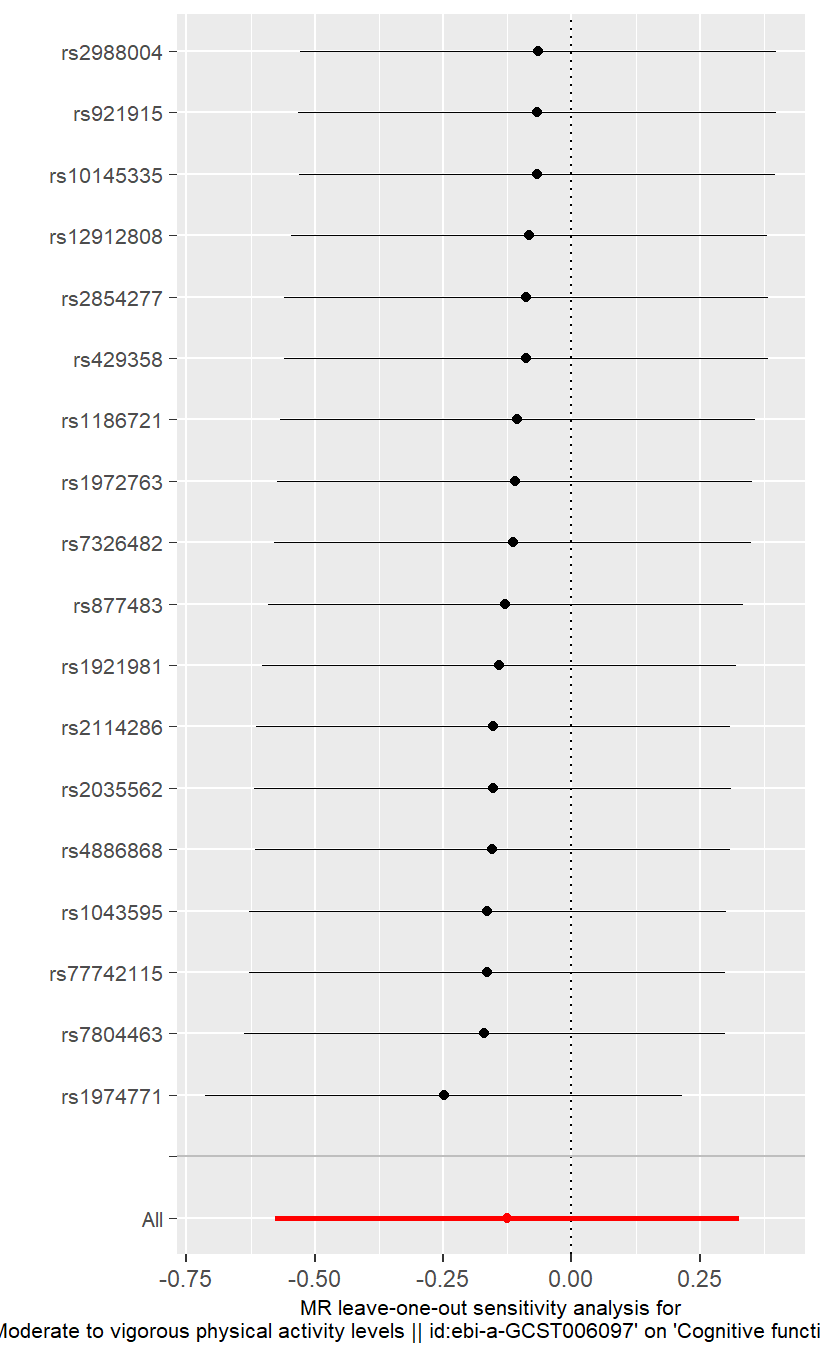


**Supplementary Figure S4.** Leave-one-out sensitivity analysis of moderate to vigorous physical activity on cognitive function under genome-wide significance threshold instrumental variables.


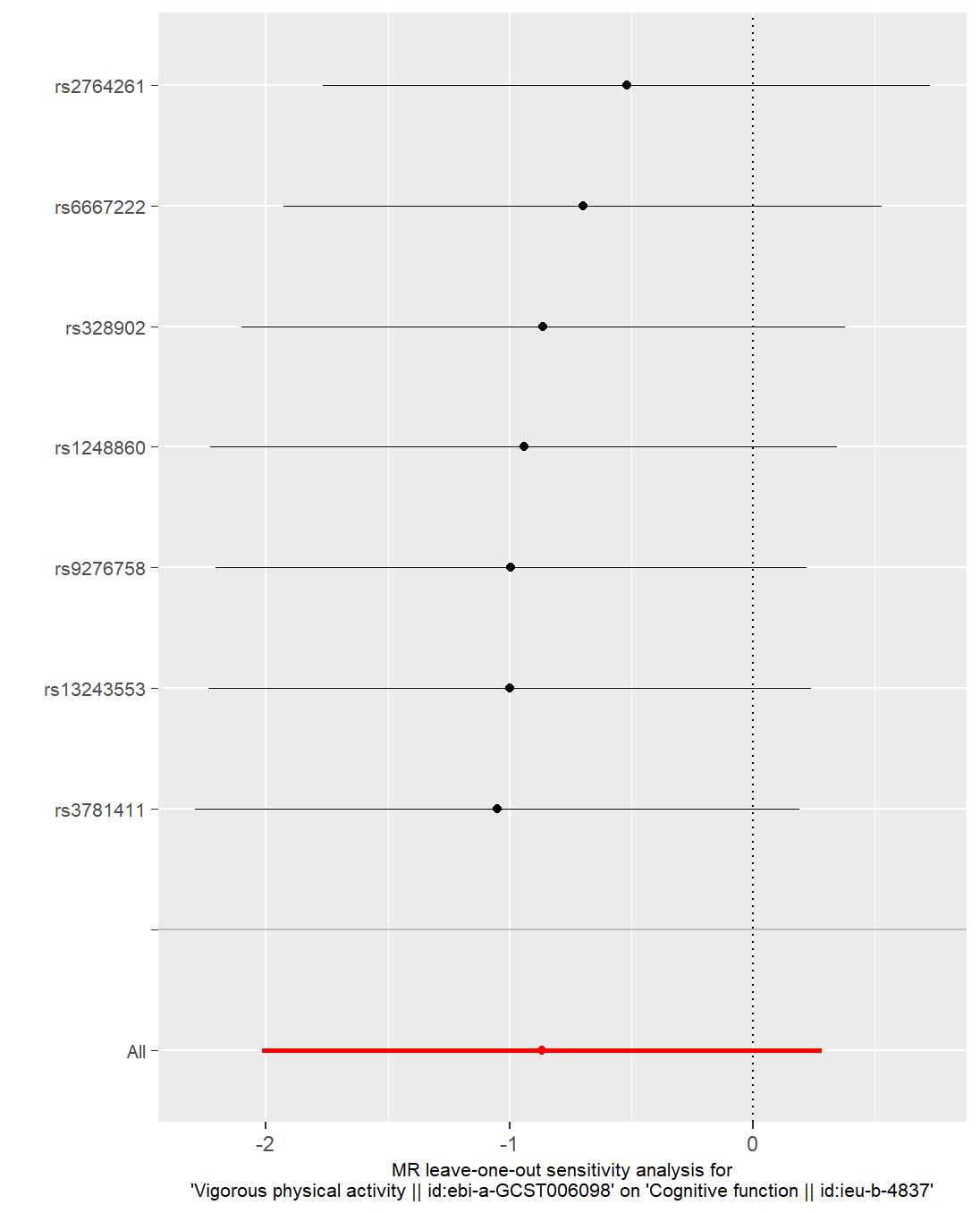


**Supplementary Figure S5.** Leave-one-out sensitivity analysis of vigorous physical activity on cognitive function under genome-wide significance threshold instrumental variables.


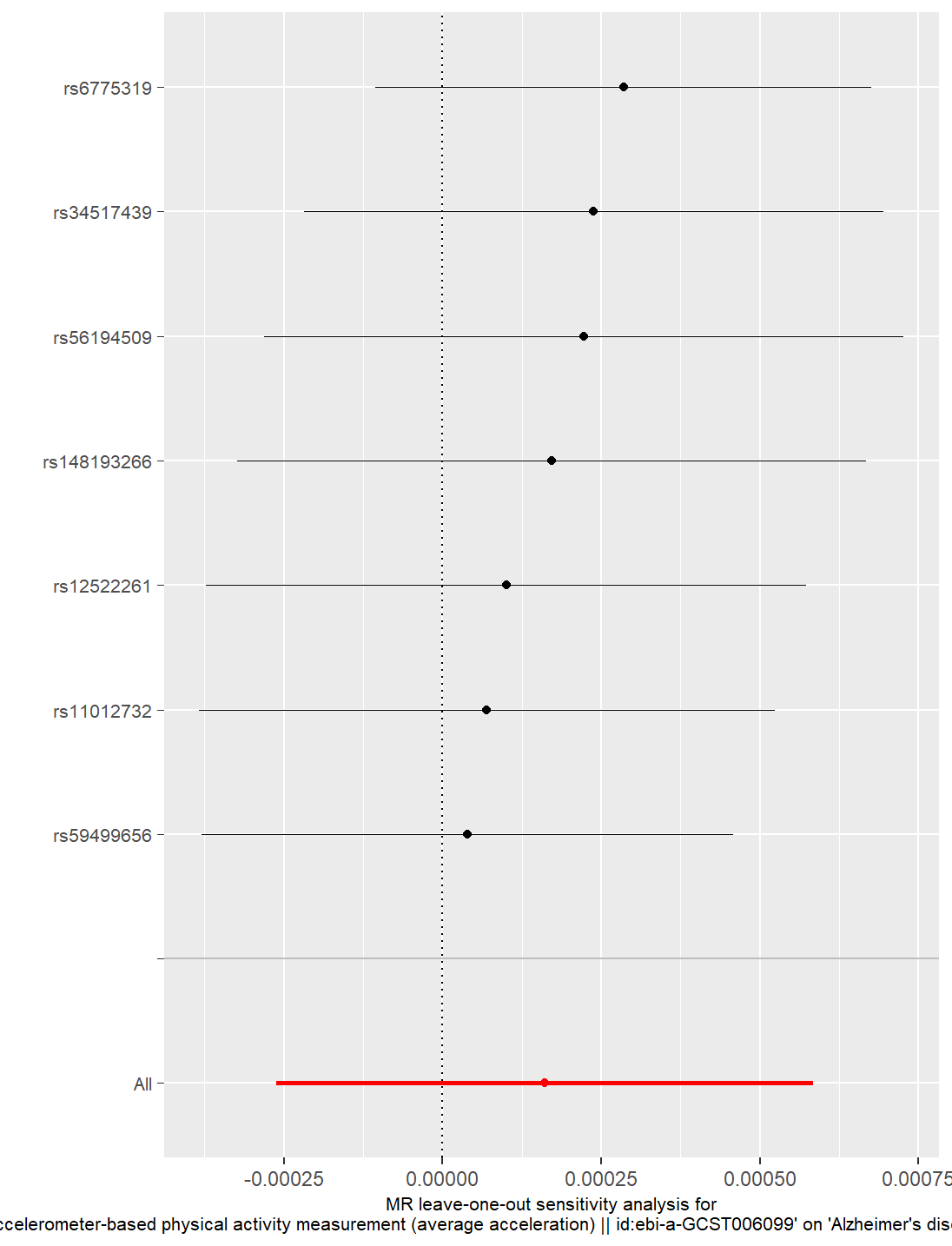


**Supplementary Figure S6.** Leave-one-out sensitivity analysis of average physical activity on Alzheimer's disease under genome-wide significance threshold instrumental variables.


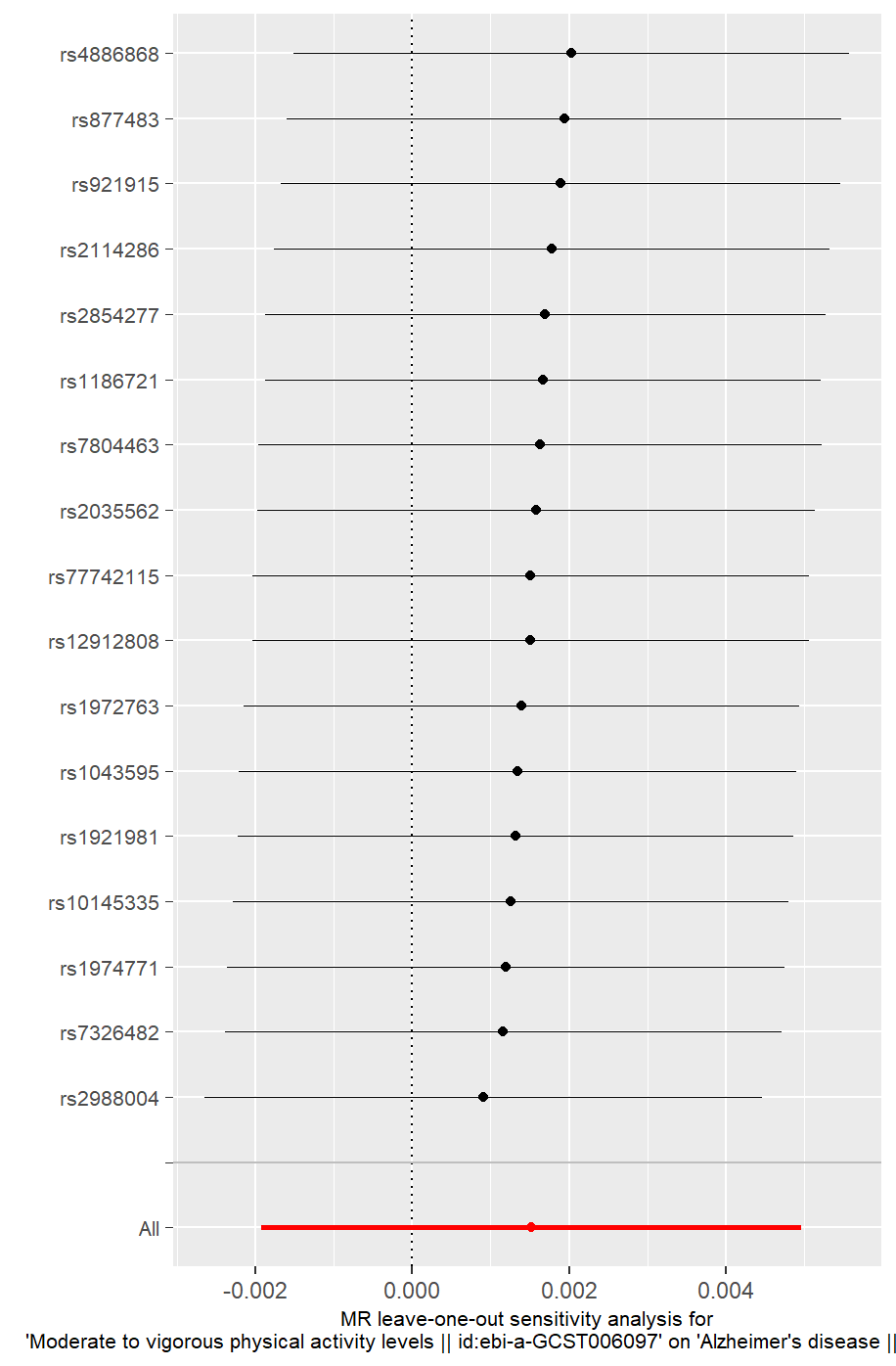


**Supplementary Figure S7.** Leave-one-out sensitivity analysis of moderate to vigorous physical activity on Alzheimer's disease under genome-wide significance threshold instrumental variables.


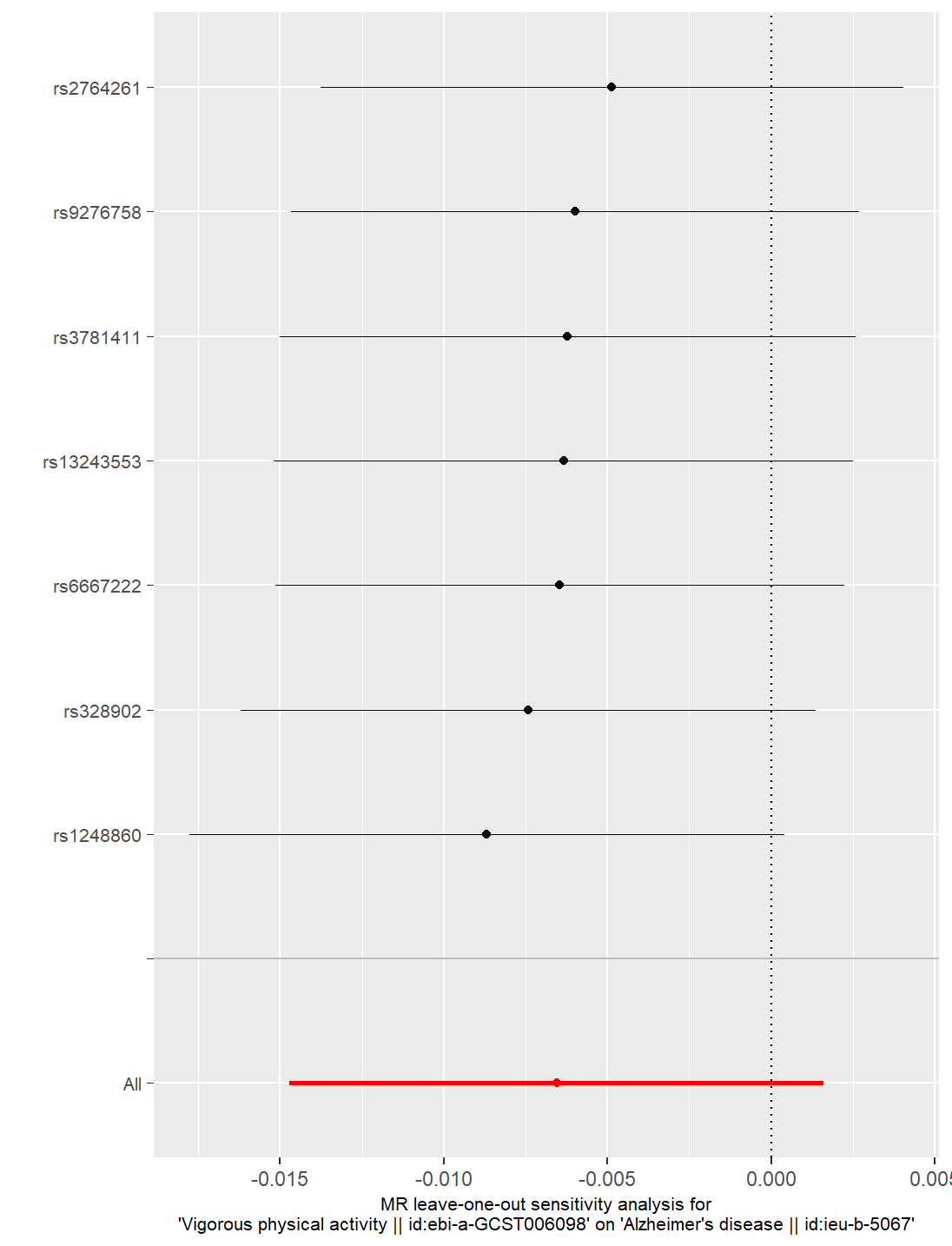


**Supplementary Figure S8.** Leave-one-out sensitivity analysis of vigorous physical activity on Alzheimer's disease under genome-wide significance threshold instrumental variables.


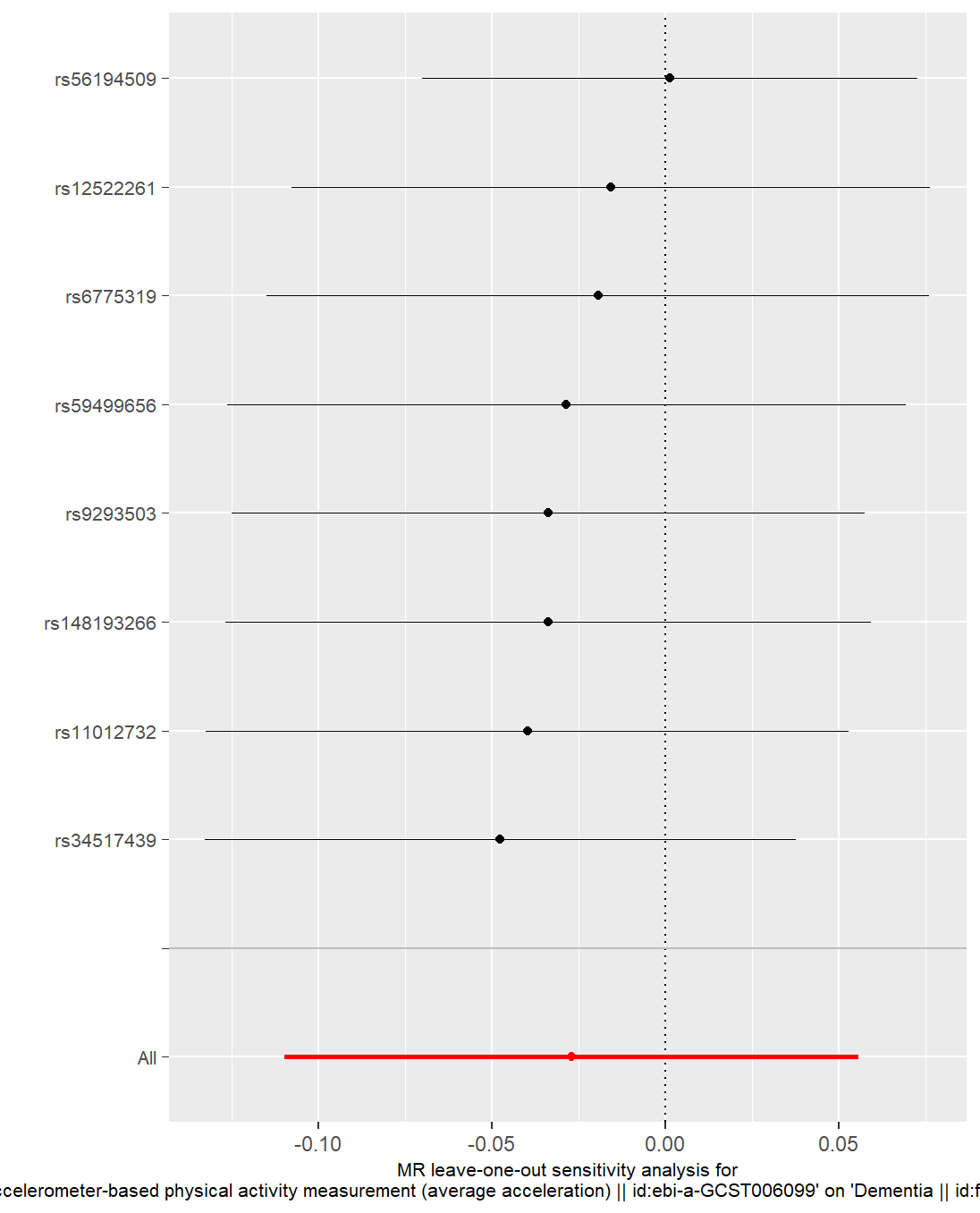


**Supplementary Figure S9.** Leave-one-out sensitivity analysis of average physical activity on dementia under genome-wide significance threshold instrumental variables.


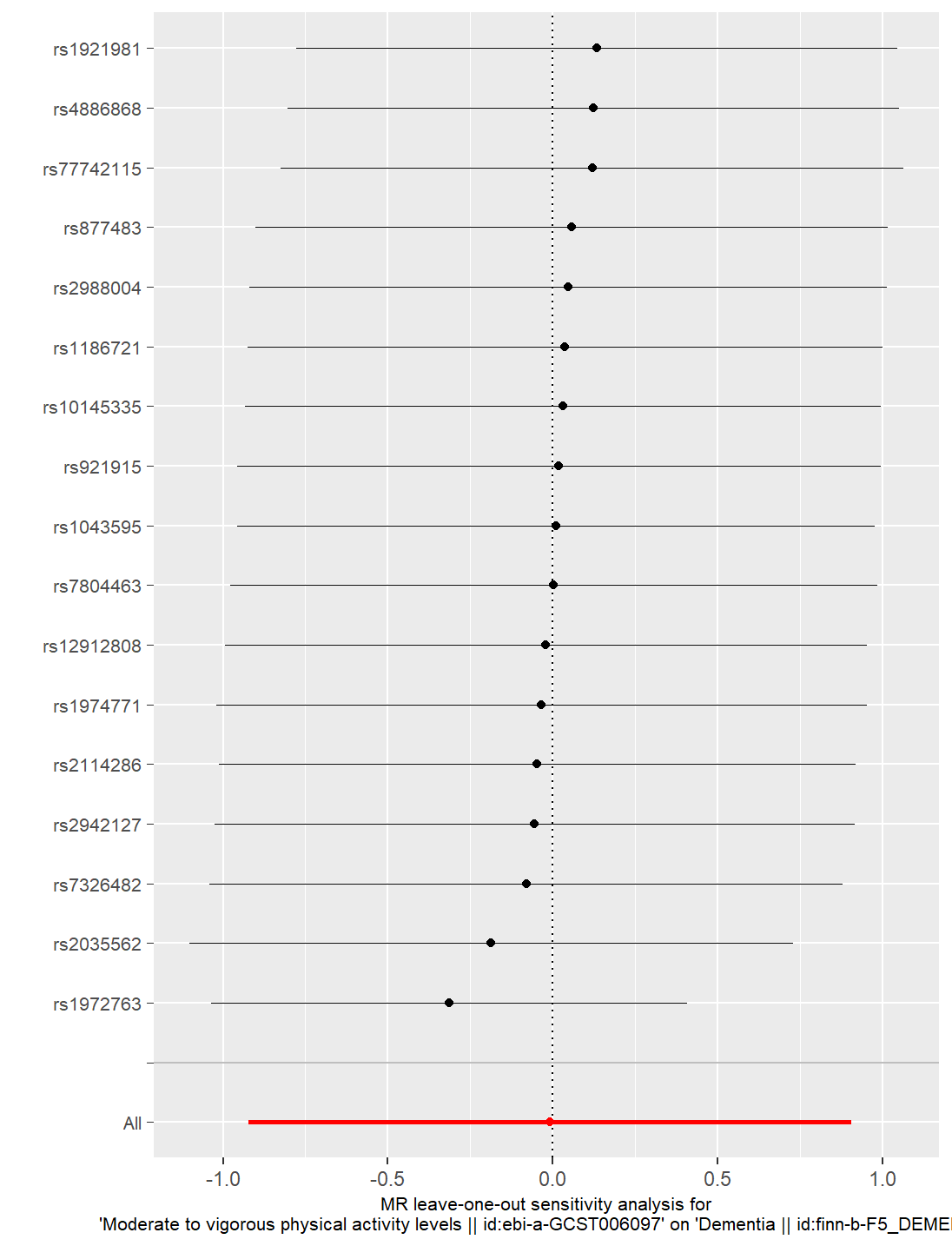


**Supplementary Figure S10.** Leave-one-out sensitivity analysis of moderate to vigorous physical activity on dementia under genome-wide significance threshold instrumental variables.


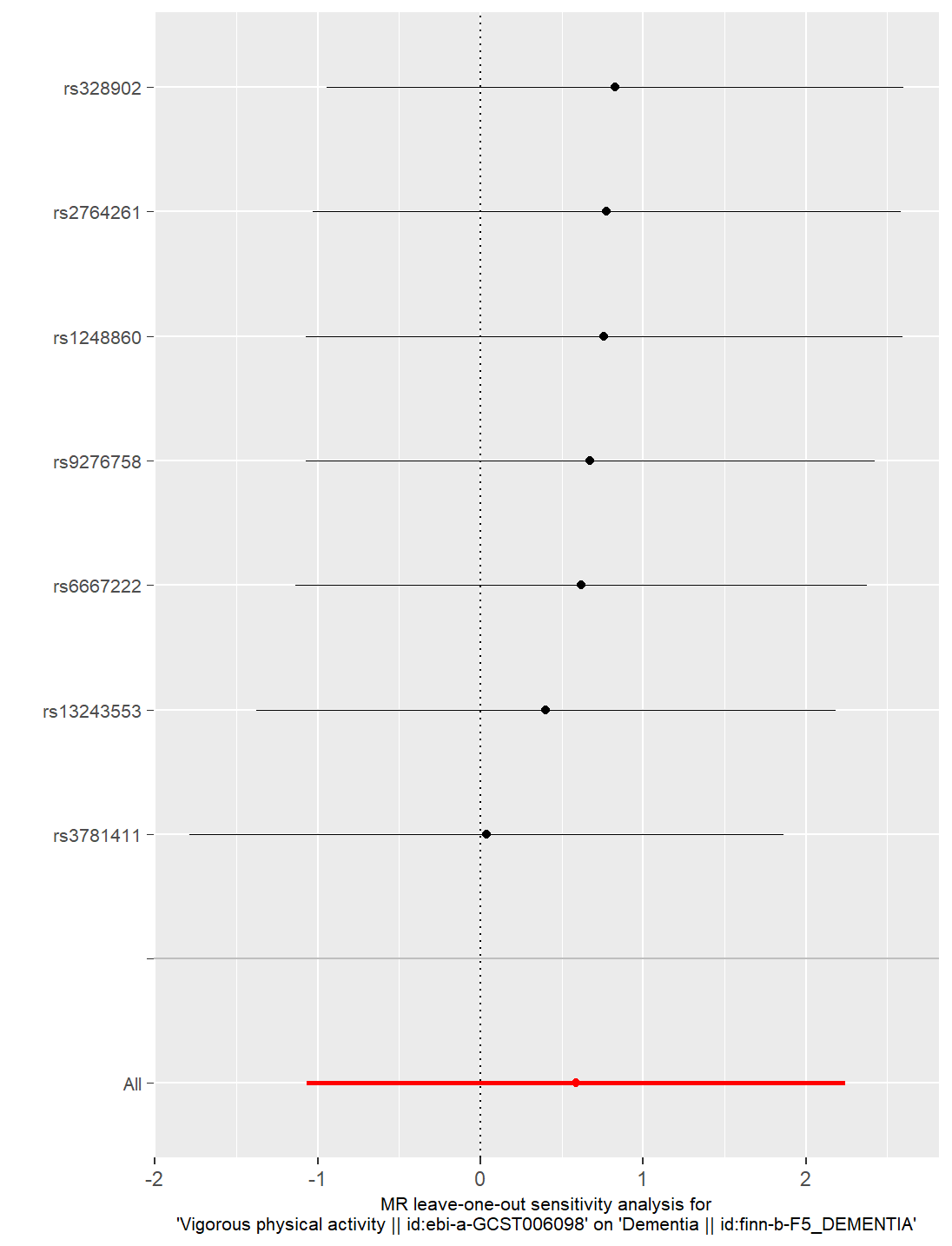


**Supplementary Figure S11.** Leave-one-out sensitivity analysis of vigorous physical activity on dementia under genome-wide significance threshold instrumental variables.
